# Semi-stochastic models for *Salmonella* infection within finishing pig units in the UK^☆^

**DOI:** 10.1016/j.mbs.2013.06.004

**Published:** 2013-10

**Authors:** Alexander D.C. Berriman, Damian Clancy, Helen E. Clough, Robert M. Christley

**Affiliations:** a Institute of Infection & Global Health, Department of Epidemiology & Population Health, University of Liverpool, Leahurst Campus, Chester High Road, Neston, Cheshire CH64 7TE, UK; b Department of Mathematical Sciences, University of Liverpool, Peach Street, Liverpool L69 7ZL, UK

**Keywords:** Semi-stochastic model, Basic reproduction number, *R*_0_, *Salmonella*

## Abstract

A multi-group semi-stochastic model is formulated to describe *Salmonella* dynamics on a pig herd within the UK and assess whether farm structure has any effect on the dynamics. The models include both direct transmission and indirect (via free-living infectious units in the environment and airborne infection). The basic reproduction number $R_{0}$ is also investigated. The models estimate approximately 24.6% and 25.4% of pigs at slaughter weight will be infected with *Salmonella* within a slatted-floored and solid-floored unit respectively, which corresponds to values found in previous abattoir and farm studies, suggesting that the model has reasonable validity. Analysis of the models identified the shedding rate to be of particular importance in the control of *Salmonella* spread, a finding also evident in an increase in the $R_{0}$ value.

## Highlights

• We model *Salmonella* transmission around varying structures of pig farm within the UK.
• We formulate and analyse the basic reproduction number for this system.
• Identify the shedding rate to be of particular importance in *Salmonella* spread.
• Highlight differences in *Salmonella* dynamics between differing farm structures.

## 1 Introduction

*Salmonella* control on farm is extremely important as *Salmonella* species are a major cause of zoonotic disease. Pork, after eggs and poultry, is considered to be a principal source of human food-borne infections. In the United Kingdom (UK) 10,071 confirmed cases of human salmonellosis were reported in 2009 [1], however the true number of cases is unknown. It is unclear how many cases are directly a result of pork and pork products, however in Denmark, pork was estimated to have caused between 11.5–19.1% of human salmonellosis cases in 2004 [2]. As field studies are expensive, the development of theoretical methods to analyse on-farm control of *Salmonella* is warranted. Investigating mechanisms that drive *Salmonella* transmission is important, as such information can help inform the development of control strategies.

An abattoir study in 2003 showed 23.4% (${\mathrm{CI}}_{95}$ 19.9–27.3; [3]) of pigs were *Salmonella* positive, with the most common serovar being *Salmonella* Typhimurium (≈70% of incidents; [4]), which shows very little change from previous studies [5]. As the last stage of the pig’s life cycle is the ‘finishing’ stage, it is likely that this part of the system poses the biggest risk to public health. The finishing stage of production involves the fattening of pigs up to slaughter weight. Typically this is done on a grower-finisher farm that rears pigs from approximately 6 to 23 weeks of age. The models developed here focus on this stage of the production system.

A number of studies have previously developed models describing *Salmonella* transmission around various types of pig unit [6–9], using a discrete time modelling approach. This approach is arguably unrealistic for such a system, as events unfold continuously. These models use a number of different categories with regard to *Salmonella* status, generally with regard to infection status. In the simplest form animals are classed as susceptible, shedding or carrying [6,7]. However Lurette et al. [8] includes seronegative shedding, seropositive shedding and seropositive carrying animals. Within Soumpasis et al. [9], infectious animals are differentiated into high infectious or low infectious categories. The models of Hill et al. and Soumpasis et al. [6,9] include an immune state. Only Ivanek et al. [7] includes a latent period, which presumably is excluded from other studies due to its short duration. The study by Lurette et al. [8] also incorporates infection via environmental contamination, by applying a dose effect function. Environmental contamination is updated depending on the numbers of bacteria shed, serological status of the animal and at every cleaning and disinfection.

A ‘typical’ structure of a pig unit with regard to management practice is difficult to define, due to the varying nature of practices adopted between farms. However, there are generally 2 structures of building used, which have varying flooring styles; a fully-slatted unit and solid floored unit, both of which are described in [10].

The overall purpose of this study is to describe *Salmonella* dynamics on a finishing unit in the UK and assess whether farm structure has any effect on *Salmonella* dynamics. To this end, we develop stochastic simulation models reflecting the 2 different structures of pig unit, using continuous time Markov chains.

## 2 Methods

### Slatted-floored unit

We use the term ‘slatted unit’ to refer to a pig farm that has slatted flooring, and consequently faeces shed can fall through the slats. Within the model, a room is made up of 5 pens on either side of a corridor, i.e., 5 pens on row 1 and 2, so a total of 10 pens within a room; a building contains 4 rooms. Animals are classed as susceptible (S), infectious (I), carrying (C) or recovered and immune (R) with regard to their *Salmonella* status. The infected class has been differentiated to include animals that are infectious (i.e., shedding, and therefore capable of passing on the infection) and carrying (infected but not shedding), as such a state has been identified in the literature [11,12]. An infectious pig is assumed to be infected within the gut, and a carrier pig is assumed to be an animal that carries the bacteria internally but is not capable of passing on infection. A carrier pig may return to the infectious state before eventually becoming recovered, and a recovered (immune) pig may return to the susceptible state. Consequently, denote the numbers of susceptible (S), infectious (I), carrying (C) and recovered (R) pigs in pen *i* of row *n* at time *t* as $X_{\mathrm{ni}}(t)$, where X=(S,I,C,R),i=1,2,…,20 and n=1,2.

**Fig. 1:**
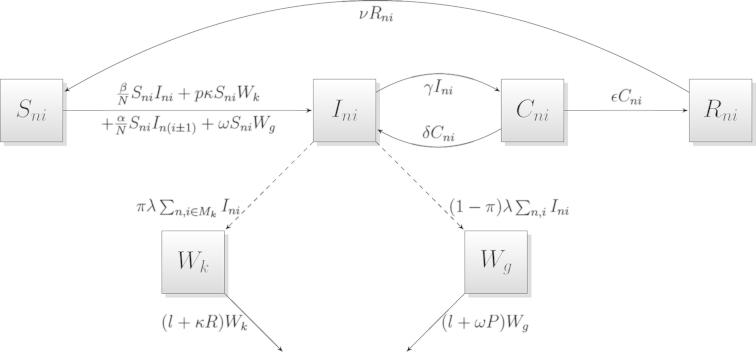
Flow diagram representing transmission routes and other processes described by Table 1. Parameters are defined in Table 2. Note: *R* denotes the number of pigs within a room, *P* denotes the number of pigs on farm and $M_{k}$ denotes the set of pens within room *k*.

The model incorporates direct host-to-host transmission, which can occur between susceptible and infectious animals within the same pen and between neighbouring pens. Additionally, infectious individuals shed infectious units (bacteria) into their local environment (i.e. within their room), a proportion of which fall through the slats and are then pooled into a general environment. Denote by $W_{k}\text{,}k=1\text{,}2\text{,}3\text{,}4$ and $W_{g}$ the number of bacteria within each local environment (room) and the number of bacteria within the general environment respectively. Hence the model also includes transmission via encounters with free-living bacteria in the local environment, which represents indirect faecal-oral transmission. Airborne transmission is also a factor within *Salmonella* transmission, which is assumed to be dependent on the number of bacteria within the general environment. A flow diagram that represents the transmission routes is shown in Fig. 1. The various transitions are represented by their corresponding transition rates as set out in Table 1.

Note that S,I,C,R and *W* are random variables taking discrete sets of values. Since the number of infectious units in the environment is enormous, comparative to herd size, and shedding and pathogen death happen frequently, these events are modelled deterministically, with $W_{1}\text{,}W_{2}\text{,}W_{3}\text{,}W_{4}\text{,}W_{g}$ being represented as continuous-valued quantities. That is, we use a semi-stochastic approximation to the model described in Table 1, following [21]. The algorithm for simulating the process is described in detail in Appendix A.

Values for many demographic parameters are derived from the literature [10,13–20]. As *Salmonella* Typhimurium has been the most common serovar isolated in pigs over a number of years [4,5], all parameters (where possible) were chosen in an attempt to reflect the dynamics of this specific serovar. Estimates for some parameters which are related to epidemiology are not yet available. All parameter values are given in Table 2. Full discussion of our choices of parameter values appears in chapter 4 of [22].

Various assumptions have been made in order to reduce the complexity of the model. It is assumed that the farm operates on an all-in-all-out basis (as opposed to continuous flow); that is, pigs enter and leave the unit in batches and therefore enter and leave the unit as a group. It is assumed that pigs are weaned elsewhere and then grown through to finishing in the same building. Consequently, pigs are received at approximately 7 weeks of age and finished to slaughter weight; approximately 23 weeks of age. It is also assumed that pigs remain in the same pen until they reach slaughter weight, i.e. there is no mixing of pigs. Furthermore, a constant number of pigs are present (i.e., no mortality) and pigs are the only source of infection.

Within an infected herd, there is an associated *Salmonella* prevalence amongst pigs entering the unit. Although animal prevalence varies greatly, on average in the UK approximately 17% of weaners entering a unit are infected [23]. As such, each animal entering the unit has a 15% chance of being infectious, and a 5% chance of being a carrier. The initial *Salmonella* status of the animals is randomly assigned upon entering the unit.

### Solid-floored unit

Another style of unit used within the UK is a solid-floored unit. In order to account for this change in farm structure, various modifications to the model must be made. Within a solid-floored unit (structure described by MLC [10]) 2 rows of pens lie centrally within a building, with a solid division between the rows. A scraping passage is used for cleaning that runs along each row of pens. We take the number of pens to be identical to that used within the slatted model in order to ensure a direct comparison can be made. However, there are not multiple rooms within this style of unit, due to the style of cleaning that is needed on farm.

This model does not involve multiple bacterial environments, but rather 1 common environment. Within this environment, there is an associated number of bacteria, denoted by $W_{g}$, which can result in infection via consumption or via airborne infection. A major difference between the 2 models is the way in which the bacteria within the environment develop over time. It is assumed that the unit is cleaned efficiently on a weekly basis. As such, when the unit is cleaned, the number of bacteria within the environment is instantaneously reduced by a proportion (1-q), where *q* is the probability of surviving the cleaning and disinfection process. Other than cleaning, other transitions and their rates are as set out in Table 1. As for the slatted unit, we actually employ a semi-stochastic model in which $W_{g}$ is treated as a continuous-valued deterministic process.

## 3 Results

### Slatted unit

**Fig. 2:**
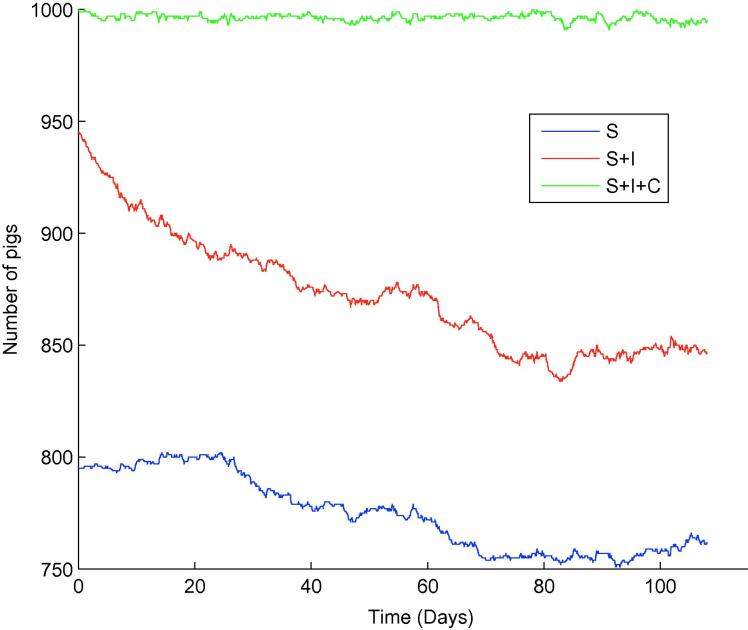
One typical iteration showing the cumulative totals of susceptible, infectious and carrying animals over time within the slatted unit. S= susceptible, I= infectious, C= carrying.

**Fig. 3:**
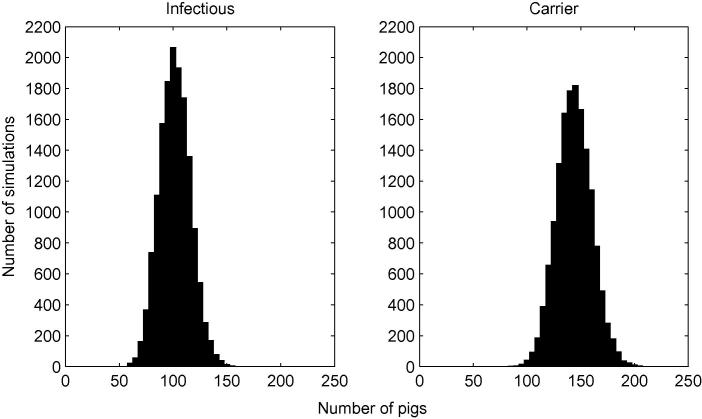
Slatted unit prevalence at slaughter. Plots appear to be approximately normally distributed, with a mean and standard deviation of 101.7 and 9.9 for infectious pigs, and 143.95 and 16.3 for carrying pigs, from 15,000 simulations.

From the slatted unit transmission model as described previously, the dynamics of *Salmonella* over time *t* can be simulated for $0⩽t⩽T_{\max}$; Fig. 2 shows one typical simulation. Rather than plotting numbers of animals in each category, Fig. 2 shows cumulative totals for S,S+I and S+I+C, in an attempt to make clearer the correspondence between types of event (as listed in Table 1) and the behaviour of the plots. The number of infectious animals initially decreases, whereas the number of carriers initially increases, before both appear to level off. Note that transitions to the recovered state are relatively rare – the duration an animal remains infected (either infectious or carrying) is thought to be quite long, and indeed the model of Lurette et al. [8] does not allow for recovery to an infection-free state at all. We also see from Fig. 2 that the number of susceptible animals declines appreciably from its initial level, so that although a large part of the prevalence at $T_{\max}$ is attributable to animals which were already infected on entry to the unit, the disease dynamics within the unit do play a significant role in determining slaughter-age prevalence. Looking specifically at slaughter-age prevalence, Fig. 3 shows results of 15,000 simulations. The mean prevalence (including both infectious and carrying animals) at $t=T_{\max}$ was found to be 24.6%, with standard deviation 2.59 (5th and 95th percentiles 20.3% and 28.8% respectively), with the majority of pigs classed as carrying (≈14.4%, standard deviation 1.63) as opposed to infectious (≈10.2%, standard deviation 1.44).

For the slatted model, we are able to calculate the basic reproduction number $R_{0}$, which is defined to be the average number of secondary infections directly produced by one infected individual introduced into a susceptible host population (for example, [24]). The relevance of $R_{0}$ as a threshold parameter is in terms of the long-term behaviour of the process - infection can only persist in the long run provided $R_{0}>1$. Consequently, in computing $R_{0}$ we do not take into account the limited time frame $0⩽t⩽T_{\max}$. Note that $R_{0}$ does not describe the time dynamics of the process, and consequently does not in itself determine whether there is a food risk, since this is dependent on the time frame of infection. An alternative would be to investigate the effective reproduction number, defined to be the average number of secondary infections resulting from one infective individual in a given population in which the infection is spreading (for example, [25]). This quantity would account for both the fact that some individuals become immune, and the limited time frame, resulting in a value which is smaller than $R_{0}$ and which changes through the course of an outbreak. $R_{0}$ is more widely used in epidemiology, being simpler to compute and in some sense reflecting more fundamental characteristics of the infection process. Rather than trying to compute effective reproduction numbers, we will instead make some allowance for the limited time frame by considering a modification of our model in which we set δ=0. The motivation for this is that with the parameter values of Table 1, very few infected pigs will have time to pass from the carrying state back into the infectious state before time $T_{\max}$. Our calculation in Appendix B of the mean effective infectious period assumes that a pig may return from the carrying to the infectious state indefinitely, which is appropriate in studying long term behaviour of the model. Setting δ=0 amounts to assuming that pigs never return from the carrying to the infectious state, which is a reasonable approximation over the restricted time frame we consider. Hence in addition to $R_{0}$ itself we consider a modified basic reproduction number ${\overset{˜}{R}}_{0}$, obtained using our $R_{0}$ calculations of Appendix B but with the effective infectious period $\frac{1}{\gamma }\left(1+\frac{\delta }{∊}\right)$ replaced by the infectious period $\frac{1}{\gamma }$.

Based on the parameters given in Table 2, the value of $R_{0}$ is 0.8204 (calculations in Appendix B), while the modified value ${\overset{˜}{R}}_{0}$ is 0.5274. As $R_{0}$ is less than 1, the usual inference is for eventual disease fade out. With the introduction of 1 infectious animal into the herd, generally the infection dies out immediately, which is consistent with the low $R_{0}$ value. However, there are a number of reasons as to why this system might take longer for the dynamics to evolve. What must be taken into account is the presence of bacteria within the environment, which persists in the environment for a long period and can be present in large quantities. As such, the presence of the bacterial environments is thought to result in extending the period of persistence of disease. The use of $R_{0}$ assumes an infinitely large population is present in each pen; although N=25 is not particularly large, it should be large enough for the calculations to be used effectively. Due to the complicated system, the presence of carrying animals appears to keep the infection sustained for a longer period of time. As there are a large number of infectious pigs entering the system at t=0, this corresponds to a large number of potential infections, and therefore, even with a low $R_{0}$, could be sufficient to sustain the infection until slaughter age.

To study this effect further, a simplified deterministic model was used to investigate long-term behaviour. This simplified model does not account for pen-to-pen transmission or airborne transmission, as the effects of these routes are thought to be small. As such, we would expect a slight underestimation of the full model prevalence. Treating the herd as homogeneously mixing, the simplified model is given by the following system of ordinary differential equations.

$$\begin{array}{cc} & \frac{\mathrm{dS}}{\mathrm{dt}}=-\beta \mathrm{SI}-p\kappa \mathrm{SW}+\nu (1-S-I-C)\text{,}\\ & \frac{\mathrm{dI}}{\mathrm{dt}}=\beta \mathrm{SI}+p\kappa \mathrm{SW}+\delta C-\gamma I\text{,}\\ & \frac{\mathrm{dC}}{\mathrm{dt}}=\gamma I-(\delta +∊)C\text{,}\\ & \frac{\mathrm{dW}}{\mathrm{dt}}=\pi \lambda I-(l+\kappa )W\text{.} \end{array}$$

**Fig. 4:**
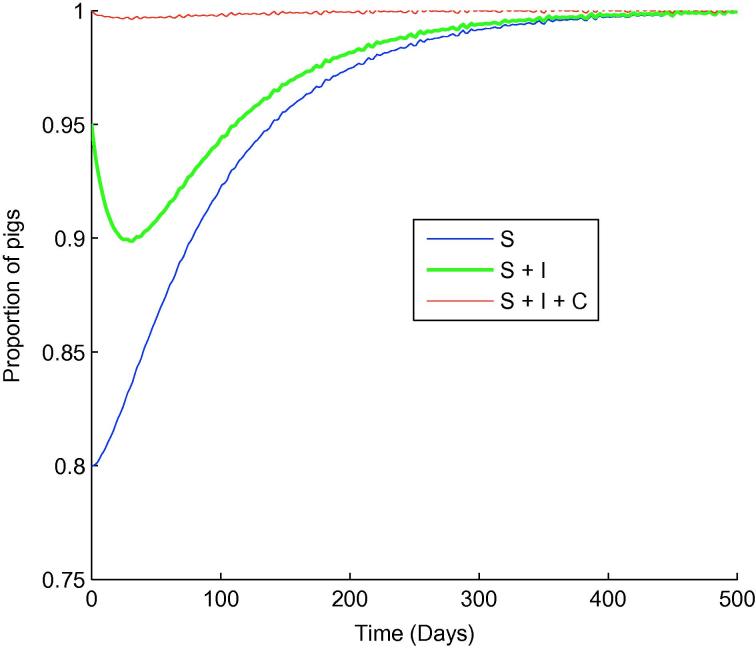
The simplified deterministic slatted unit model. S= susceptible, I= infectious, C= carrying.

The trajectory of this deterministic system (with parameter values and initial conditions as before) is shown in Fig. 4. We see that the infection does persist until slaughter age $T_{\max}=108$, in line with the semi-stochastic model, but eventually fades out, consistent with our calculated $R_{0}<1$. Note that the simplified deterministic model is sufficient to obtain a rough idea of the long-term behaviour of the model much more quickly than by semi-stochastic simulations, but that model behaviour after slaughter age $T_{\max}$ is irrelevant in practice, and the semi-stochastic model gives a more complete and reliable picture of behaviour up until $T_{\max}$.

### Solid-floored unit

**Fig. 5:**
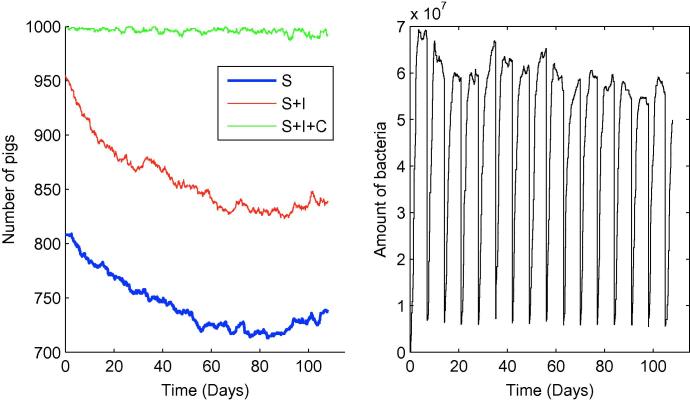
Cumulative totals of susceptible, infectious and carrying animals and environmental bacteria over time, solid unit, one typical iteration. S= susceptible, I= infectious, C= carrying.

When adapting the model to represent a solid floored unit, a mean prevalence of ≈ 25.4% with standard deviation 3.20 (5th and 95th percentiles 20.1% and 30.7% respectively) is found, with ≈ 10.0% of pigs classed as infectious (standard deviation 1.69) and ≈ 15.4% as carriers (standard deviation 1.97). The distribution of infectious and carrying animals within the solid unit is similar to that found within the slatted-floored unit (Fig. 3). A plot of a typical trajectory (Fig. 5) shows similar behaviour to the slatted model; after the initial phase the number of carriers remain consistently higher than the number of infectious pigs. The number of bacteria within the environment is found to be higher than in the slatted unit. A plot of the number of bacteria within the environment over time (Fig. 5) is shown in order to illustrate how cleaning affects the availability of bacteria. The cleaning effect means that it is not straightforward to compute $R_{0}$ for this model.

### Validation

Data from the Zoonoses Action Plan (ZAP) and Zoonoses National Control Programme (ZNCP) farm visits and results from a British abattoir study were used to validate the model. Farm visits by the Animal Health and Veterinary Laboratories Agency (AHVLA) found 31% of samples positive for *Salmonella* spp. in 2005 compared to 24% in 2009 [26]. This concurs with an abattoir study in 2003 which showed 23.4% (${\mathrm{CI}}_{95}$ 19.9–27.3; [3]) of pigs to be *Salmonella* positive at slaughter. Unfortunately data were not available for the type of unit pigs came from. As such, this prevalence must be used for both models described. Furthermore, a study in 1999–2000 [5] found *S.* Typhimurium in 11.1% of caecum samples. As all parameter values (where possible) were related to *S.* Typhimurium, an estimate from the model of shedding around this proportion provides a good estimate. Furthermore, a final prevalence was found to be within the confidence interval of on-farm studies. As such, the results from the model would be deemed reasonable.

### Shedding rate and “super-shedders”

The presence of *Salmonella* in the environment is thought to be extremely important in sustaining on-farm prevalence. As such, the number of bacteria shed in the faeces is an important factor within the spread of *Salmonella*; i.e., the shedding rate, *λ*. Within both the slatted and solid unit, a ten times higher shedding rate results in a slaughter age prevalence of 91.2% and 90.85% respectively; which potentially highlights that the on-farm prevalence was not dependent of the building structure when shedding was high.

This high *Salmonella* prevalence could be due to the presence of a number of pigs shedding high numbers of bacteria, otherwise known as “super-shedders.” Studies have shown a wide array of *Salmonella* numbers shed in pigs [11,27–29] and the existence of super-shedders in other species has been proved [30,31]. The way in which our model was formulated did not allow the analysis of individual pigs within the farm; therefore, the additional shedding could be due to a large number of pigs shedding medium levels of bacteria, or a low number of pigs shedding high numbers of bacteria. Clearly the way in which “super-shedders” have been modelled was not ideal, since it assumes the average shedding rate is increased for all animals. Nevertheless, increasing the shedding rate (*λ*) does to some extent model the presence of “super-shedders,” as the average shedding rate would see an increase. Arguably however, as the model dynamics appear to be driven by the bacterial environment, explicitly modelling “super-shedders” is not necessary.

**Fig. 6:**
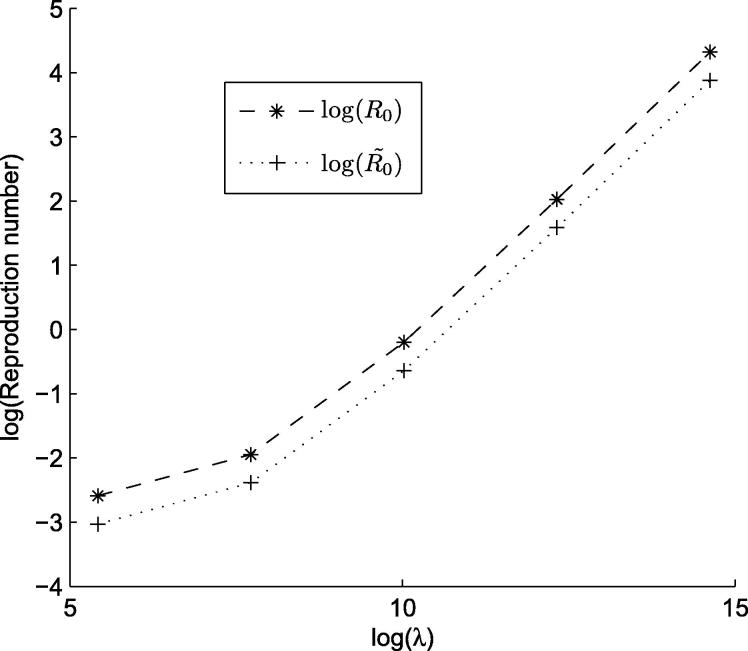
Graph highlighting the effect of the shedding rate (*λ*) on $R_{0}$ and ${\overset{˜}{R}}_{0}$. Natural logs were used, with base parameter log (*λ*) = 10.02.

When the shedding rate was increased tenfold, $R_{0}$ was found to be 7.5970, with ${\overset{˜}{R}}_{0}=4.8838$. The increase in $R_{0}$ and ${\overset{˜}{R}}_{0}$ with increased shedding rate is illustrated in Fig. 6. As a result of the increase in $R_{0}$, *Salmonella* infection would be able to spread and persist within the environment even if only a single infectious pig entered the unit initially.

**Fig. 7:**
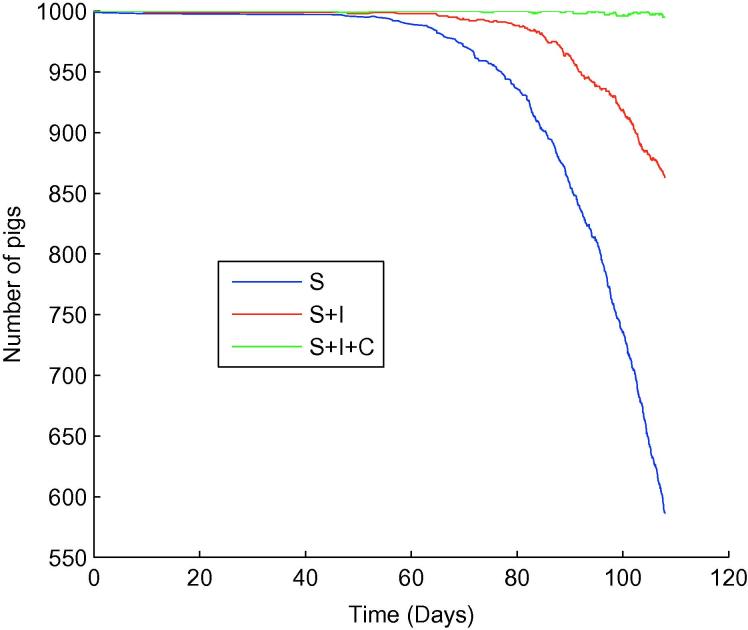
One typical iteration when 1 infectious pig, shedding high levels of *Salmonella* ($\lambda =2.25\times 10^{5}$), was introduced in an otherwise susceptible population into a slatted finishing unit. S= susceptible, I= infectious, C= carrying.

**Fig. 8:**
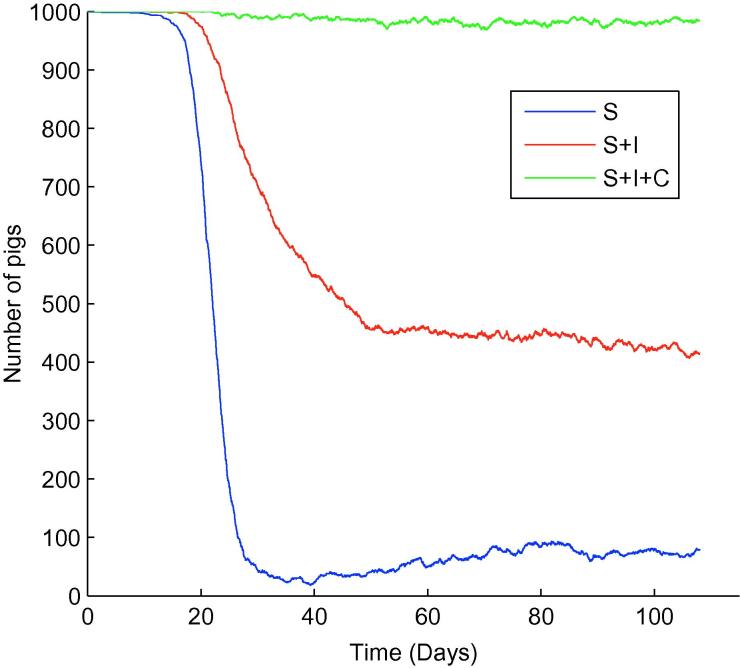
One typical iteration when 1 infectious pig, shedding high levels of *Salmonella* ($\lambda =2.25\times 10^{5}$), was introduced into an otherwise susceptible population into a solid finishing unit. S= susceptible, I= infectious, C= carrying.

When looking at an outbreak initiated by a single infectious animal, a significant difference between the farm structures emerges. For a slatted unit consisting of 1 large room (i.e. ignoring the presence of 4 rooms and therefore removing any potential influence room structure has on the dynamics), we found (Fig. 7) that infection took a long time to become established (approximately 60 days). Within the solid unit however, the dynamics were very different, with infection becoming established within 15 days (Fig. 8). Furthermore, the prevalence immediately prior to slaughter differed considerably between farm types: the slatted unit had a prevalence of approximately 58%, compared to 92% for the solid unit. As all bacteria shed were available for consumption within the solid unit, it was thought that this enabled a quicker uptake of infection, which consequently resulted in a greater slaughter age prevalence. As such, it was thought flooring type played a major role within this scenario.

## 4 Conclusion and discussion

The aim of the study was to describe the *Salmonella* dynamics on a pig finishing unit in the UK and assess whether farm structure has any effect on the dynamics.

A key finding from the study is that the basic reproduction number $R_{0}$ (for the parameter values used within Table 2) is below 1. This has consequences for intervention strategies, as a standard strategy would be to apply an intervention that would reduce $R_{0}$ below 1. It appears that a main reason for the apparent persistence of disease, despite this low $R_{0}$ value, is the number of infected animals entering the unit. As such, it could be deduced that a reduction in the number of infected animals leaving the breeder/nursery stages of production could be expected to lead to a reduction in *Salmonella* prevalence in slaughter age pigs. However, as the model does not account for external influences, this result should be viewed with caution.

For the standard parameter values given in Table 2, the dynamics appear similar between the two unit structures. A point of note is the large number of animals classed as carrying the bacteria compared to those classed as infectious. This has a number of implications for food safety as there is a large reservoir of animals that could become re-infectious upon transport to the abattoir for example. The distribution of these infected animals varies slightly between structures, whereby solid flooring is associated with a higher upper interval (37.5% compared to 35.2%), which could indicate that solid flooring is a potential risk factor for *Salmonella* infection. This is possibly be due to the presence of a larger mean number of bacteria within the environment within the solid unit compared to the slatted unit ($5.05\times 10^{7}$ and $3.51\times 10^{6}$ respectively). Furthermore, there could be an implication here that for cleaning and disinfection to be as effective as possible, it would need to be conducted frequently. Consequently, the cost effectiveness of applying such control strategies should be investigated. As the basic models have now been developed, future work can investigate interventions that obtain some form of on-farm control of *Salmonella*.

The amount of bacteria shed once a pig becomes infectious was found to be of great importance. Various studies have previously established the existence of super shedders in other animal systems (for example *Escherichia coli* O157 in cattle [30,31]), and proved that such animals have an important role in the transmission dynamics. Although such animals have not yet explicitly been proved to exist within the pig population, the distribution of *Salmonella* shedding in pigs is large [11,27–29,32]. As such, it is not unreasonable to conclude that some pigs shed higher numbers of bacteria than others, and could therefore be classed as “super-shedders.” The finding that higher shedding has a substantial effect upon $R_{0}$ (and therefore the potential presence of “super-shedders”) was important for the industry as it highlighted the need for interventions to address this issue.

Interestingly, with the average number of infected animals entering the unit, both models had a similar slaughter age *Salmonella* prevalence when shedding was high. However, the rate at which infection was able to spread varied between the models, whereby the solid model had an accelerated uptake of infection. This in turn had implications with regard to the application of an intervention. With the accelerated uptake of infection within the solid unit, the time at which an intervention should be applied in order to be as effective as possible may need to be during the initial uptake of infection. However this would require further investigation.

## Figures and Tables

**Table 1:** Transition rates for the fully stochastic models.

Event	State transition	Rate
A susceptible becomes infectious by an infective within the same pen (*ni*)	($S_{\mathrm{ni}}\text{,}I_{\mathrm{ni}}$) → ($S_{\mathrm{ni}}-1\text{,}I_{\mathrm{ni}}+1$)	$\frac{\beta }{N}S_{\mathrm{ni}}I_{\mathrm{ni}}$
An infective in pen *ni* ceases to infect but remains carrying *Salmonella*	($I_{\mathrm{ni}}\text{,}C_{\mathrm{ni}}$) → ($I_{\mathrm{ni}}-1\text{,}C_{\mathrm{ni}}+1$)	$\gamma I_{\mathrm{ni}}$
A carrier in pen *ni* starts reinfecting	($I_{\mathrm{ni}}\text{,}C_{\mathrm{ni}}$) → ($I_{\mathrm{ni}}+1\text{,}C_{\mathrm{ni}}-1$)	$\delta C_{\mathrm{ni}}$
A carrier in pen *ni* recovers	($C_{\mathrm{ni}}\text{,}R_{\mathrm{ni}}$) → ($C_{\mathrm{ni}}-1\text{,}R_{\mathrm{ni}}+1$)	$∊C_{\mathrm{ni}}$
A recovered pig in pen *ni* becomes re-susceptible	($S_{\mathrm{ni}}\text{,}R_{\mathrm{ni}}$) → ($S_{\mathrm{ni}}+1\text{,}R_{\mathrm{ni}}-1$)	$\nu R_{\mathrm{ni}}$
An infectious pig from a neighbouring pen (n(i±1))^a^ infects a susceptible in pen *ni*	($S_{\mathrm{ni}}\text{,}I_{\mathrm{ni}}$) → ($S_{\mathrm{ni}}-1\text{,}I_{\mathrm{ni}}+1$)	$\frac{\alpha }{N}S_{\mathrm{ni}}I_{n(i\pm 1)}$
Indirect transmission via the airborne route	($S_{\mathrm{ni}}\text{,}I_{\mathrm{ni}}\text{,}W_{g}$) → ($S_{\mathrm{ni}}-1\text{,}I_{\mathrm{ni}}+1\text{,}W_{g}-1$)	$\omega S_{\mathrm{ni}}W_{g}$

*Slatted unit*		
Indirect transmission from bacterial consumption, within room k^b^	($S_{\mathrm{ni}}\text{,}I_{\mathrm{ni}}\text{,}W_{k}$) → ($S_{\mathrm{ni}}-1\text{,}I_{\mathrm{ni}}+1\text{,}W_{k}-1$)	$p\kappa S_{\mathrm{ni}}W_{k}$
Bacteria are shed into the general environment	($W_{g}$) → ($W_{g}+1$)	$(1-\pi )\lambda \sum_{n\text{,}i}I_{\mathrm{ni}}$
Bacteria are shed into the local (room) environment	($W_{k}$) → ($W_{k}+1$)	$\pi \lambda \sum_{n\text{,}i\in M_{k}}I_{\mathrm{ni}}$
Death of bacteria	($W_{x})$^c^ → ($W_{x}-1$)	${\mathrm{lW}}_{x}$
Consumption of bacteria that does not result in transmission	($W_{k})$ → ($W_{k}-1$)	$\kappa (\sum_{n\text{,}i\in k}(I_{\mathrm{ni}}+C_{\mathrm{ni}}+R_{\mathrm{ni}}+(1-p)S_{\mathrm{ni}}))W_{k}$

*Solid unit*		
Indirect transmission from bacterial consumption	($S_{\mathrm{ni}}\text{,}I_{\mathrm{ni}}\text{,}W_{g}$) → ($S_{\mathrm{ni}}-1\text{,}I_{\mathrm{ni}}+1\text{,}W_{g}-1$)	$p\kappa S_{\mathrm{ni}}W_{g}$
Bacteria shed into the environment	($W_{g}$) → ($W_{g}+1$)	$\lambda \sum_{n\text{,}i}I_{\mathrm{ni}}$
Death of bacteria	($W_{g}$) → ($W_{g}-1$)	${\mathrm{lW}}_{g}$
Consumption of bacteria that does not result in transmission	($W_{g}$) → ($W_{g}-1$)	$\kappa (\sum_{n\text{,}i}(I_{\mathrm{ni}}+C_{\mathrm{ni}}+R_{\mathrm{ni}}+(1-p)S_{\mathrm{ni}}))W_{g}$

*Note:* Only state elements that are affected by the corresponding event are shown. The full set of state elements is $\left\{(S_{\mathrm{ni}}\text{,}I_{\mathrm{ni}}\text{,}C_{\mathrm{ni}}\text{,}R_{\mathrm{ni}}):n=1\text{,}2\text{;}i=1\text{,}2\ldots \text{,}\mathrm{PensPerSide}\right\}$, $\left(W_{g}\text{,}W_{1}\text{,}W_{2}\text{,}W_{3}\text{,}W_{4}\right)$.

a if n(i±1) in same room as *ni*.

b for k=1,2,3,4.

c where x∈{1,2,3,4,g}.

**Table 2:** Definitions of the parameters used in the model.

Parameter	Definition (units)	Parameter estimate	Reference
*N*	Number of pigs per pen	25	[10]
*PensPerSide*	Number of pens on either side of a corridor	20	[10]
*β*	Infection rate	Assume $1.67\times 10^{-3}$	–
γ	The rate at which a pig ceases to remain infectious (day^−1^)	$\frac{1}{26}$ = 0.03846	[13,14]
δ	The rate at which a carrier becomes re-infectious (day^−1^)	$\frac{1}{108}$ = 0.00926	–
∊	The rate at which a pig ceases to carry the bacteria (day^−1^)	$\frac{1}{60}$ = 0.01667	[14]
ν	Loss of immunity (day^−1^)	0.5	–
*λ*	Shedding rate (cfu day^−1^)	$2.25\times 10^{4}$	[15,16]
κ	Proportion of cfu present ingested (day^−1^): Slatted	$4.23\times 10^{-4}$	[17,16]
	Solid	$3.17\times 10^{-5}$	
*l*	Bacteria death rate (day^−1^)	$\frac{1}{84}$ = 0.01190	[18]
*p*	Probability of infection from bacterial consumption	$2.30\times 10^{-6}$	[19,13]
*α*	Cross infection rate	Assume $1.14\times 10^{-6}$	–
*π*	Proportion of faeces that remains in a room	0.4	–
*q*	Proportion of faeces that remains present after cleaning	0.1	–
$T_{\max}$	Time spent in unit (days)	108	[20]
ω	Airborne infection rate	Assume $1.02\times 10^{-14}$	–

## Appendix A

The definition of the fully stochastic model is given in Table 1. We use a semi-stochastic model in which, because of the very different time scales, the dynamics of the free-living stages (bacteria) are treated deterministically in simulation. The sum of the rates of all events affecting the pig population is given by

$$\mathrm{TotalRate}(t)=\frac{\beta }{N}\underset{n\text{,}i}{\sum }S_{\mathrm{ni}}I_{\mathrm{ni}}+\gamma \underset{n\text{,}i}{\sum }I_{\mathrm{ni}}+\nu \underset{n\text{,}i}{\sum }R_{\mathrm{ni}}+\delta \underset{n\text{,}i}{\sum }C_{\mathrm{ni}}+∊\underset{n\text{,}i}{\sum }C_{\mathrm{ni}}+\frac{\alpha }{N}\underset{n\text{,}i}{\sum }S_{\mathrm{ni}}\underset{j\in V_{i}}{\sum }I_{\mathrm{nj}}+p\kappa \underset{n\text{,}i}{\sum }S_{\mathrm{ni}}W_{k(i)}+\omega \underset{n\text{,}i}{\sum }S_{\mathrm{ni}}W_{g}$$

where $k(i)=1+\left[\frac{i-1}{5}\right]$ with $\left[\frac{i-1}{5}\right]$ denoting integer part, and $V_{i}=\{i-1\text{,}i+1\}\cap \{j:k(j)=k(i)\}$. That is, k(i) labels the room containing pen *i*, and $V_{i}$ is the set of pens adjacent to pen *i* and contained within the same room. Between transitions affecting the pig population, numbers of bacteria evolve deterministically according to the differential equations

$$\begin{array}{cc} & \frac{{\mathrm{dW}}_{k}}{\mathrm{dt}}=\pi \lambda \underset{n\text{,}i\in M_{k}}{\sum }I_{\mathrm{ni}}-\left(l+\kappa R\right)W_{k}\;k=1\text{,}2\text{,}3\text{,}4\text{,}\\ & \frac{{\mathrm{dW}}_{g}}{\mathrm{dt}}=(1-\pi )\lambda \underset{n\text{,}i}{\sum }I_{\mathrm{ni}}-(l+\omega P)W_{g}\text{,} \end{array}$$

where $M_{k}$ denotes the set of pens within room *k*.

Given the current state of the pig population, the differential equations for $\left(W_{1}\text{,}W_{2}\text{,}W_{3}\text{,}W_{4}\text{,}W_{g}\right)$ may be solved explicitly, and hence the greatest possible value of TotalRate(t) is calculated, denoted *UpperTR*. The time to the next event is then simulated using the Poisson thinning algorithm [33, Section 11.5]. That is, a proposed next event time *T* is generated from the exponential distribution with mean 1/UpperTR. With probability TotalRate(T)/UpperTR this proposal is accepted; otherwise the procedure is restarted from time *T*, a new proposed event time is generated, and so on until an acceptance occurs. Once an event time has been accepted, the nature of the event is chosen at random, with non-bacterial event probabilities proportional to the rates in Table 1 and bacterial values (*W*) evaluated at the event time.

## Appendix B

The basic reproduction number $R_{0}$ is calculated for the slatted model. One way of calculating the basic reproduction number is by calculating the next generation matrix, as described by Diekmann and Heesterbeek [34] and Heffernan et al. [35]. The elements of this matrix consist of the expected number of secondary infections due to a single primary infection in a fully susceptible population, calculated class by class [36]. The value of $R_{0}$ is given by the dominant eigenvalue of the next generation matrix [34].

The mean effective infectious period, following one infection, is:

$$\frac{1}{\gamma }\left(1+\frac{\delta }{\delta +∊}+{\left(\frac{\delta }{\delta +∊}\right)}^{2}+\cdots \right)=\frac{1}{\gamma }\left(1+\frac{\delta }{∊}\right)\text{.}$$

During this period, an infective in pen *ni* makes contacts in pen *ni* at rate *β*, and in pens n(i-1) and n(i+1) at rate *α*, iff n(i±1) is within the same room as *ni*. Furthermore, bacteria are shed at rate *λ*. Denote by $D_{\mathrm{ni}\text{,}\mathrm{mj}}$ the average number of direct infectious contacts to pen *mj* made by an infective in pen *ni* during one effective infectious period. Then

$$D_{\mathrm{ni}\text{,}\mathrm{ni}}=\frac{\beta }{\gamma }\left(1+\frac{\delta }{∊}\right)\text{,}D_{\mathrm{ni}\text{,}n(i-1)}=D_{\mathrm{ni}\text{,}n(i+1)}=\frac{\alpha }{\gamma }\left(1+\frac{\delta }{∊}\right)\;\text{if}\;n(i\pm 1)\;\text{is within the same room as}\;\mathrm{ni}\text{,}D_{\mathrm{ni}\text{,}\mathrm{mj}}=0\;\text{otherwise.}$$

The mean number of bacteria shed by one infective is $\frac{\lambda }{\gamma }\left(1+\frac{\delta }{∊}\right)$, where a proportion (*π*) remains available for consumption (local environment) and the remainder (1-π) is under the slats (general environment) and affects the airborne route of transmission. Each bacterium lives for an average time of $\frac{1}{l+R\kappa }$ and $\frac{1}{l+P\omega }$, within the local and general environments respectively, where *R* is the number of pigs within a room and *P* is the total number of pigs present. During this time, infectious contacts in pen *mj* are made at rates pNκ and Nω, respectively. Therefore, the average number of indirect contact events arising from one infected individual during a single effective infectious period is $\frac{\lambda }{\gamma }\left(1+\frac{\delta }{∊}\right)\pi \frac{\mathrm{pN}\kappa }{l+R\kappa }+\frac{\lambda }{\gamma }\left(1+\frac{\delta }{∊}\right)(1-\pi )\frac{N\omega }{l+P\omega }$.

The entries of the next generation matrix, *M*, are therefore:

$$\begin{array}{cc} & M_{\mathrm{ni}\text{,}\mathrm{ni}}=\frac{\beta }{\gamma }\left(1+\frac{\delta }{∊}\right)+\frac{\lambda }{\gamma }\left(1+\frac{\delta }{∊}\right)\left[\pi \frac{\mathrm{pN}\kappa }{l+R\kappa }+(1-\pi )\frac{N\omega }{l+P\omega }\right]\text{,}\\ & M_{\mathrm{ni}\text{,}n(i\pm 1)}=\frac{\alpha }{\gamma }\left(1+\frac{\delta }{∊}\right)+\frac{\lambda }{\gamma }\left(1+\frac{\delta }{∊}\right)\left[\pi \frac{\mathrm{pN}\kappa }{l+R\kappa }+(1-\pi )\frac{N\omega }{l+P\omega }\right]\;\text{provided}\;i\pm 1\;\text{is in the same room as}\;i\text{,}\\ & M_{\mathrm{ni}\text{,}\mathrm{mj}}=\frac{\lambda }{\gamma }\left(1+\frac{\delta }{∊}\right)\left[\pi \frac{\mathrm{pN}\kappa }{l+R\kappa }+(1-\pi )\frac{N\omega }{l+P\omega }\right]\;\text{otherwise.} \end{array}$$
